# Identification of hub ferroptosis-related genes and immune infiltration in lupus nephritis using bioinformatics

**DOI:** 10.1038/s41598-022-23730-8

**Published:** 2022-11-05

**Authors:** Weitao Hu, Xiaoqing Chen

**Affiliations:** Department of Rheumatology, The Second Affiliated Hospital of Fujian Medical University, 34 North Zhongshan Road, Licheng District, Quanzhou, 362000 Fujian People’s Republic of China

**Keywords:** Nephrology, Rheumatology

## Abstract

Lupus nephritis (LN) is one of the most severe and more common organ manifestations of the autoimmune disease, systemic lupus erythematosus. Ferroptosis, a novel type of programmed cell death, so far its role in LN remains uncertain. In the present study, we explored the role of ferroptosis in LN and its relationship with the immune response. The GSE112943 LN dataset was downloaded from the Gene Expression Omnibus database. Ferroptosis-Related Genes (FRGs) that drive, suppress or mark ferroptosis were retrieved from the public FerrDb database. The gene expression matrix of the GSE112943 dataset was analyzed with the “limma” package in R to obtain differentially expressed genes (DEGs) between LN and healthy samples. Subsequently, the crossover genes between DEGs and FRGs were identified as differentially expressed ferroptosis-related genes (DE-FRGs). Protein–protein interaction (PPI) network analysis, visualization, and identification of hub lupus nephritis ferroptosis-related genes (LN-FRGs) were performed with STRING and Cytoscape, while their Gene Ontology (GO) and Kyoto Encyclopedia of Genes and Genomes (KEGG) pathways were determined with the clusterProfiler package. Immune cell infiltration was calculated with CIBERSORT. The relationship between hub LN-FRGs and immune-infiltrated cells in LN was determined by Pearson correlation. A total of 96 DE-FRGs and 8 hub LN-FRGs (*KRAS, PIK3CA, EGFR, MAPK14, SRC, MAPK3, VEGFA,* and *ATM*) were identified. GO and KEGG functional classification indicated these genes enrichment in apoptotic process, programmed cell death, autophagy-animal, FoxO signaling pathway, relaxin signaling pathway, and VEGF signaling pathway. Infiltration matrix analysis of immune cells showed abundant Monocytes and M0/M1/M2 macrophages in LN kidney tissues. Correlation analysis revealed 8 hub LN-FRGs associated with immune-infiltrated cells in LN. In summary, overproduction of ROS and abnormal infiltration of immune cells would be implicated in the LN caused by ferroptosis. 8 hub lupus nephritis ferroptosis-related genes (LN-FRGs) which might be good biomarkers of ferroptosis in LN were identified in this study. These findings point to the immune response playing an important role in LN caused by ferroptosis via mutual regulation between hub LN-FRGs and immune-infiltrated cells.

## Introduction

Lupus nephritis (LN) is one of the most severe and more common organ manifestations of the autoimmune disease, systemic lupus erythematosus (SLE), as well as a form of glomerulonephritis^1^. Over the past decades, the understanding of the pathogenesis and characteristics of LN has improved considerably. However, LN remains the leading cause of morbidity and mortality in patients with SLE, despite greater understanding and more targeted treatment protocols^2,3^. LN is commonly treated with immunosuppressive drugs, such as glucocorticoids and cyclophosphamide or mycophenolate mofetil (MMF). Unfortunately, conventional immunosuppressive therapy is not effective in all patients, moreover, the toxic side effects of the drugs should not be underestimated^4–6^. Therefore, early & accurate diagnosis and identification of targeted therapeutic targets are crucial for LN.

Cell death has been suggested as a critical contributor to the pathogenesis of LN^7^. There is growing evidence that programmed cell death, including apoptosis, NETosis, and autophagy aggravates the progression of LN within their effects^8–10^. In recent years, Ferroptosis, a novel type of programmed cell death, has begun to be increasingly studied. Ferroptosis is driven by lethal lipid peroxidation (the result of an imbalance in cellular metabolism and redox homeostasis) and can be inhibited either by direct blockade of lipid peroxidation or by depletion of iron, in addition to growing evidence of a potential physiological role for ferroptosis in tumor suppression and immunity^11^. Cellular studies have shown that both *DPEP1* and *CHMP1A* are important regulators of a single pathway (ferroptosis) and contribute to the development of kidney disease by altering cellular iron transport^12^. One study found that *GPX4* dysfunction reduced the sensitivity of mice to acute tubular necrosis (ATN) during acute kidney injury (AKI), while Nec-1f. was found to be a solid inhibitor of *RIPK1* and a weak inhibitor of ferroptosis^13^. Zhang et al. found that ferroptosis contributes to the progression of Autosomal dominant polycystic kidney disease (ADPKD) and that ferroptosis inhibitors may be a new strategy for the treatment of ADPKD^14^. Mitophagy is a key cellular homeostatic mechanism that is activated early during AKI, and there is an important link between Mitophagy and ferroptosis^15^. However, so far it seems that the role of ferroptosis in LN has never been mentioned.

To investigate whether ferroptosis is involved in the progression of LN, we collected microarray data from the GEO database of kidney tissue from LN patients and healthy individuals. Subsequently, we screened LN differentially expressed genes (DEGs) and hub genes associated with ferroptosis by bioinformatics methods and performed immuno-infiltration analysis to analyze their correlation. In summary, there were 96 Differentially Expressed Ferroptosis-Related Genes (DE-FRGs), 8 hub lupus nephritis ferroptosis-related genes (LN-FRGs) that were identified as potential ferroptosis-related target biomarkers for LN.

## Materials and methods

### Data collection and acquisition

The Gene Expression Omnibus (GEO; www.ncbi.nlm.nih.gov/geo/)^16^ database was used to search gene microarray data of LN kidney samples. The GSE112943^17^ dataset, containing 7 healthy (control group) and 14 LN (experimental group) kidney tissue samples were selected. Probes were converted to gene symbols according to the GPL10558 platform (Illumina HumanHT-12 V4.0 expression beadchip). Ferroptosis-Related Genes (FRGs) that drive, suppress, or mark ferroptosis were retrieved from the public FerrDb database (http://www.zhounan.org/ferrdb^18^. The final 258 FRGs obtained after removing duplicate genes were used for subsequent analysis.

### Identification of differentially expressed ferroptosis-related genes (DE-FRGs)

The gene expression matrix of the GSE112943 dataset was analyzed with the “limma” package in R to obtain DEGs between LN and healthy samples. Briefly, |log2 fold change (FC)|> 1 and *P* < 0.05 were set as the selection criteria for DEGs. Subsequently, the crossover genes between DEGs and FRGs were identified as differentially expressed ferroptosis-related genes (DE-FRGs).

### Protein–protein interaction (PPI) network construction and module analysis

Interactions between different DE-FRGs were analyzed using the STRING database (http://string-db.org/^19^. PPI networks were constructed and visualized using Cytoscape software 3.9.1. (http://cytoscape.org/. ^20^ The most significant module was identified with the Cytoscape plug-in Molecular Complex Detection (MCODE) (version 2.0), which is used to identify densely connected regions by clustering a given network based on its topology^21^. Using the cytoHubba plugin, the overlap of the top 20 genes based on algorithms such as MCC, maximum neighborhood component (MNC), DNMC, Closeness, Degree, and edge percolated component (EPC) algorithms were identified as hub lupus nephritis ferroptosis-related genes (LN-FRGs).

### Functional enrichment of DE-FRGs and hub LN-FRGs

The clusterProfiler package in R was used to identify the Gene Ontology (GO) and Kyoto Encyclopedia of Genes and Genomes (KEGG)^22–24^ pathways characterizing DE-FRGs and hub LN-FRGs, as well as to explore their potential biological processes, cellular components, molecular functions, and important signaling pathways. A minimum gene set of 5 and a maximum gene set of 5000 were chosen. *P* < 0.05 and false detection rate < 0.1 were considered statistically significant.

### Evaluation of subtype distribution among immune-infiltrated cells

CIBERSORT was shown to transform a normalized gene expression matrix into the composition of 22 immune cell types based on a deconvolution algorithm^25^. Here, CIBERSORT was employed to calculate the composition of immune cells in LN and healthy samples. The algorithm employed the LM22 signature and 1000 permutations. Given *P* < 0.05, 14 LN and 7 healthy samples were selected for further analysis.

### Correlation and differential analysis of immune-infiltrated cells

For the assessment of the correlation between different immune cells, Pearson correlation coefficient was obtained from the sample data screened by CIBERSORT, *P* < 0.05. A rank sum test was used to compare the LN group with the control group.

### Correlation between hub LN-FRGs and immune-infiltrated cells in LN

Pearson correlation matrix analysis was performed on the GSE112943 immune infiltrating cell profile analyzed by CIBERSORT and the gene expression profile of this dataset. Correlations between hub LN-FRGs and immune infiltrating cells were determined using Pearson correlation coefficients (r) > 0.6 and *P* < 0.05.

## Results

### Identification of DE-FRGs in LN

**Figure 1:**
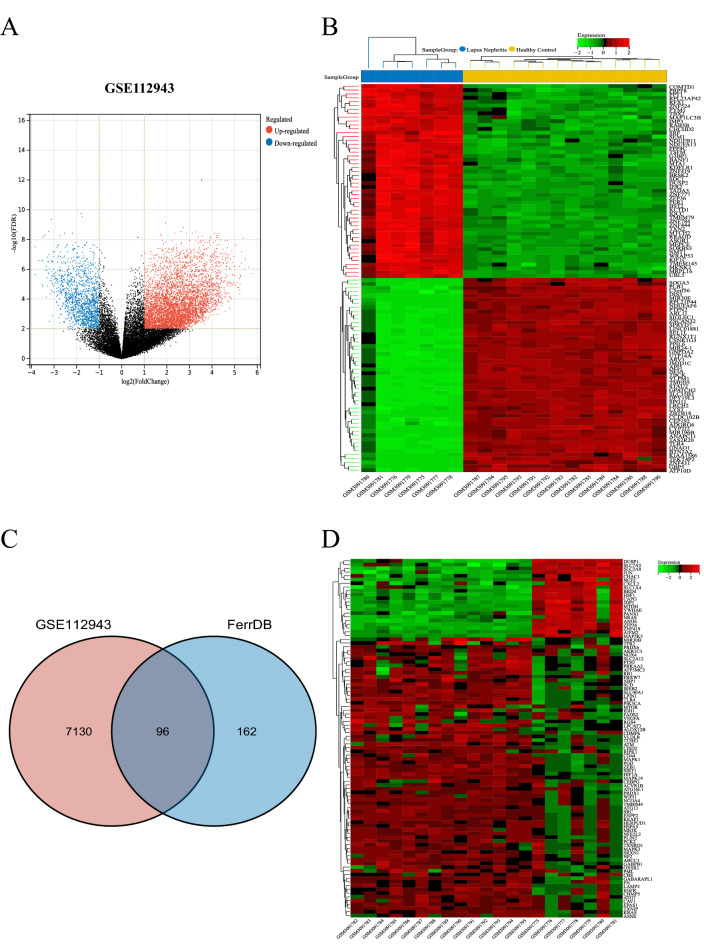
Identification of Differentially Expressed Ferroptosis-Related Genes (DE-FRGs). (**A**) Volcano plots displaying significantly differentially expressed genes. |Log2FC|≥ l and *P* < 0.05 were chosen as filtering conditions. Blue dots represent significantly downregulated genes, red dots represent significantly upregulated genes, and black dots denote genes not showing any significant difference. (**B**) Heatmap displaying the expressions of the DEGs in GSE112943. Green corresponds to lower gene expression and red to higher gene expression. (**C**) Venn diagram displaying the DE-FRGs. (**D**) Heatmap displaying the expressions of the 96 DE-FRGs in LN dataset GSE112943.

ThE GSE112943 dataset containing gene expression profiles of 7 healthy and 14 LN kidney biopsy tissue samples was retrieved from the GEO database. After standardization of microarray results, 7226 DEGs were identified; they included 5671 upregulated and 1555 downregulated genes (Fig. 1A,B). To explore FRGs differentially expressed in the LN, 258 FRGs were retrieved from FerrDb, a database of regulators, markers, and diseases involved in ferroptosis. A total of 96 FRGs were identified as DE-FRGs after taking the crossover of DEGs and FRGs (Fig. 1C). The expression of the 96 DE-FRGs in the dataset GSE112943 was shown in Fig. 1D.

### PPI network construction, module analysis, and hub LN-FRGs identification

**Figure 2:**
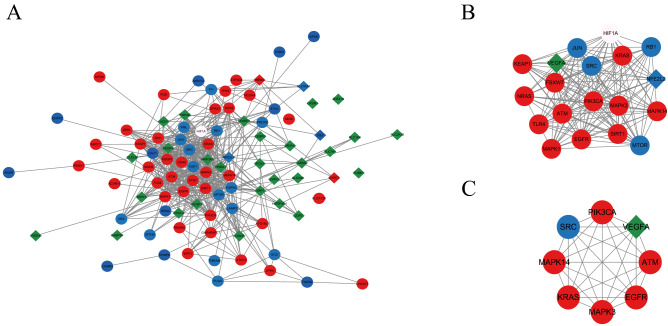
PPI network construction and module analysis. (**A**) PPI network of DE-FRGs (96 nodes and 511 edges). (**B**) Cytoscape-based identification of the densest connected regions (19 nodes and 146 edges) in the PPI network. (**C**) Hub LN-FRGs identified by 6 algorithms of cytoscape plugin cytoHubba. Red circle indicates the driver of ferroptosis, blue circle represents the suppressor of ferroptosis, green diamond indicates the marker of ferroptosis, red diamond represents both driver and marker of ferroptosis, blue diamond indicates both suppressor and marker of ferroptosis, white circle indicates the both driver and suppressor of ferroptosis.

PPI analysis of DE-FRGs was based on the STRING database and results were visualized using Cytoscape (Fig. 2A). The MCODE plug-in identified the most densely connected regions (19 nodes and 146 edges) in the PPI network (Fig. 2B). With the 6 algorithms of the Cytoscape plugin cytoHubba, we calculated the top 20 genes (Table 1). Subsequently, the top 8 intersecting genes analyzed based on the MCC, DNMC, MNC, Degree, Closeness, and EPC algorithms were selected as the hub LN-FRGs, which included *KRAS, PIK3CA, EGFR, MAPK14, SRC, MAPK3, VEGFA,* and *ATM* (Fig. 2C). Notably, all these hub LN-FRGs were upregulated in patients with LN. The details about the 8 hub LN-FRGs were shown in Table 2.

**Table 1:** The top 20 hub genes rank in cytoHubba.

MCC	DNMC	MNC	Degree	EPC	Closeness	Overlap
ATM	ATM	ATG7	ATG7	ATM	ATG7	ATM
EGFR	BRD4	ATM	ATM	CAV1	ATM	EGFR
FBXW7	CAV1	CAV1	CAV1	EGFR	CAV1	KRAS
HIF1A	CD44	EGFR	EGFR	FBXW7	EGFR	MAPK14
HSPA5	EGFR	HIF1A	HIF1A	HIF1A	HIF1A	MAPK3
JUN	FBXW7	HSPA5	HSPA5	HSPA5	HSPA5	PIK3CA
KEAP1	KRAS	JUN	JUN	JUN	JUN	SRC
KRAS	MAPK14	KEAP1	KEAP1	KEAP1	KEAP1	VEGFA
MAPK1	MAPK3	KRAS	KRAS	KRAS	KRAS	
MAPK14	NF2	MAPK1	MAPK1	MAPK1	MAPK1	
MAPK3	NOX4	MAPK14	MAPK14	MAPK14	MAPK14	
MTOR	NRAS	MAPK3	MAPK3	MAPK3	MAPK3	
NFE2L2	PIK3CA	MTOR	MTOR	MTOR	MTOR	
NRAS	RB1	NFE2L2	NFE2L2	NFE2L2	NFE2L2	
PIK3CA	SRC	PIK3CA	PIK3CA	PIK3CA	PIK3CA	
RB1	TXNIP	SIRT1	SIRT1	RB1	SIRT1	
SIRT1	VEGFA	SRC	SRC	SIRT1	SRC	
SRC	WIPI1	TLR4	TLR4	SRC	TLR4	
TLR4	YWHAE	TXNRD1	TXNRD1	TLR4	VEGFA	
VEGFA	ZEB1	VEGFA	VEGFA	VEGFA	XBP1

**Table 2:** The top 8 hub genes and their functions.

Gene Symbol	Description	Function
VEGFA	vascular endothelial growth factor A	Growth factor active in angiogenesis, vasculogenesis and endothelial cell growth. Induces endothelial cell proliferation, promotes cell migration, inhibits apoptosis and induces permeabilization of blood vessels
ATM	ATM serine/threonine kinase	Serine/threonine protein kinase which activates checkpoint signaling upon double strand breaks (DSBs), apoptosis and genotoxic stresses such as ionizing ultraviolet A light (UVA), thereby acting as a DNA damage sensor
PIK3CA	phosphatidylinositol-4,5-bisphosphate 3-kinase catalytic subunit alpha	PIP3 plays a key role by recruiting PH domain-containing proteins to the membrane, including AKT1 and PDPK1, activating signaling cascades involved in cell growth, survival, proliferation, motility and morphology
EGFR	epidermal growth factor receptor	Receptor tyrosine kinase binding ligands of the EGF family and activating several signaling cascades to convert extracellular cues into appropriate cellular responses
KRAS	GTPase KRAS	Ras proteins bind GDP/GTP and possess intrinsic GTPase activity. Plays an important role in the regulation of cell proliferation
MAPK3	Mitogen-activated protein kinase 3,namly ERK1	Serine/threonine kinase which acts as an essential component of the MAP kinase signal transduction pathway
MAPK14	Mitogen-activated protein kinase 14, namely p38	Serine/threonine kinase which acts as an essential component of the MAP kinase signal transduction pathway. MAPK14 is one of the four p38 MAPKs which play an important role in the cascades of cellular responses evoked by extracellular stimuli such as proinflammatory cytokines or physical stress leading to direct activation of transcription factors
SRC	Proto-oncogene tyrosine-protein kinase SRC	Non-receptor protein tyrosine kinase which is activated following engagement of many different classes of cellular receptors including immune response receptors, integrins and other adhesion receptors, receptor protein tyrosine kinases, G protein- coupled receptors as well as cytokine receptors

### Functional Enrichment of DE-FRGs and hub LN-FRGs

**Figure 3:**
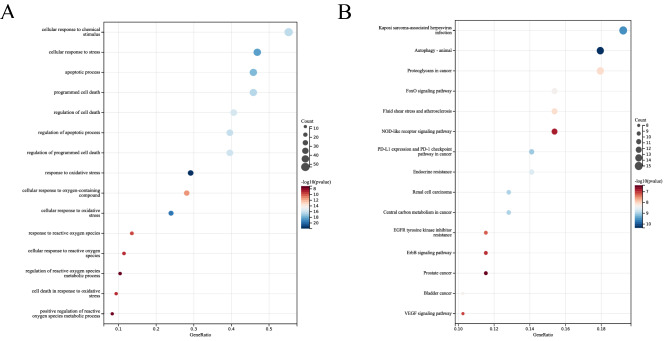
GO and KEGG enrichment analysis of DE-FRGs related to LN. (**A**) Bubble plot of enriched GO terms showing DE-FRGs. (**B**) Bubble plot of enriched KEGG pathways showing DE-FRGs. A darker color and a larger bubble denote a more significant difference.

**Figure 4:**
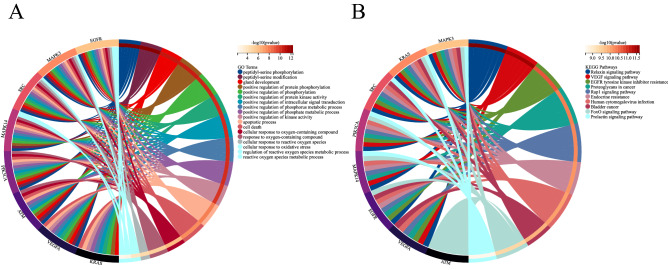
GO and KEGG enrichment analysis of Hub LN-FRGs related to LN. (**A**) GO term of Hub LN-FRGs. (**B**) KEGG enrichment of Hub LN-FRGs. The genes are linked to their assigned pathway terms via colored ribbons and are ordered according to the observed log10 *P* value, which is displayed in descending intensity of red squares next to the selected genes.

To predict the biological function of DE-FRGs, we performed functional enrichment analysis. GO analysis revealed that DE-FRGs were enriched mainly in cellular response to chemical stimulus, apoptotic process, programmed cell death, response to oxidative stress and response to reactive oxygen species (ROS) (Fig. 3A); whereas KEGG pathway analysis indicated DE-FRGs were significantly enriched in Autophagy-animal, proteoglycans in cancer, FoxO signaling pathway, and NOD-like receptor signaling pathway (Fig. 3B). GO analysis indicated that hub LN-FRGs were significantly enriched in peptidyl-serine phosphorylation, apoptotic process, cell death, cellular response to reactive oxygen species and regulation of reactive oxygen species metabolic process (Fig. 4A); whereas KEGG pathway analysis indicated hub LN-FRGs were significantly enriched in relaxin signaling pathway, VEGF signaling pathway, and EGFR tyrosine kinase inhibitor resistance (Fig. 4B). The results of the functional enrichment analysis obtained for either A or B showed that these LN genes are associated with programmed cell death or ROS.

### Distribution of immune-infiltrated cells

**Figure 5:**
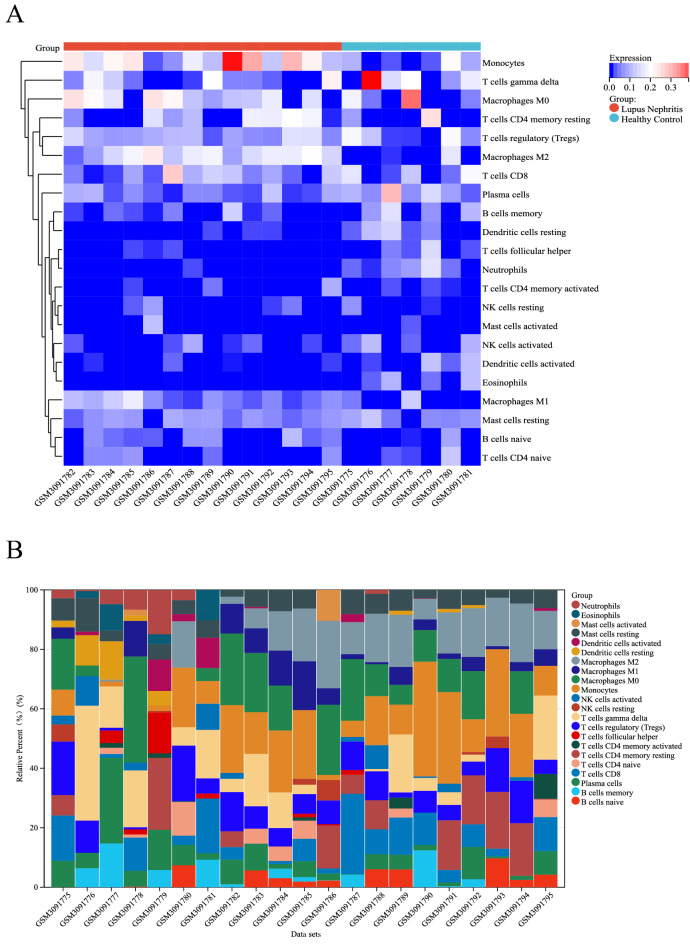
Infiltration of immune-associated cells in healthy and LN samples. (**A**) Immune cell content in each sample. (**B**) Relative percentage of 22 subpopulations of immune cells in 21 samples from the GSE112943 dataset.

The microarray was screened using the CIBERSORT inverse convolution method with *P* < 0.05, resulting in 7 healthy kidney tissues and 14 LN kidney tissue groups on the heatmap. Monocytes, M0 macrophages, M1 macrophages, and M2 macrophages were all more abundant in kidney tissues of patients with LN than in healthy controls, while T cells follicular helper, Dendritic cells resting, Eosinophils, and Neutrophils were less expressed in the kidney tissue of LN patients than in the healthy control group (Fig. 5A). Figure 5B details the distribution of 22 immune cells in each sample.

### Correlation and differential analysis of immune-infiltrated cells

**Figure 6:**
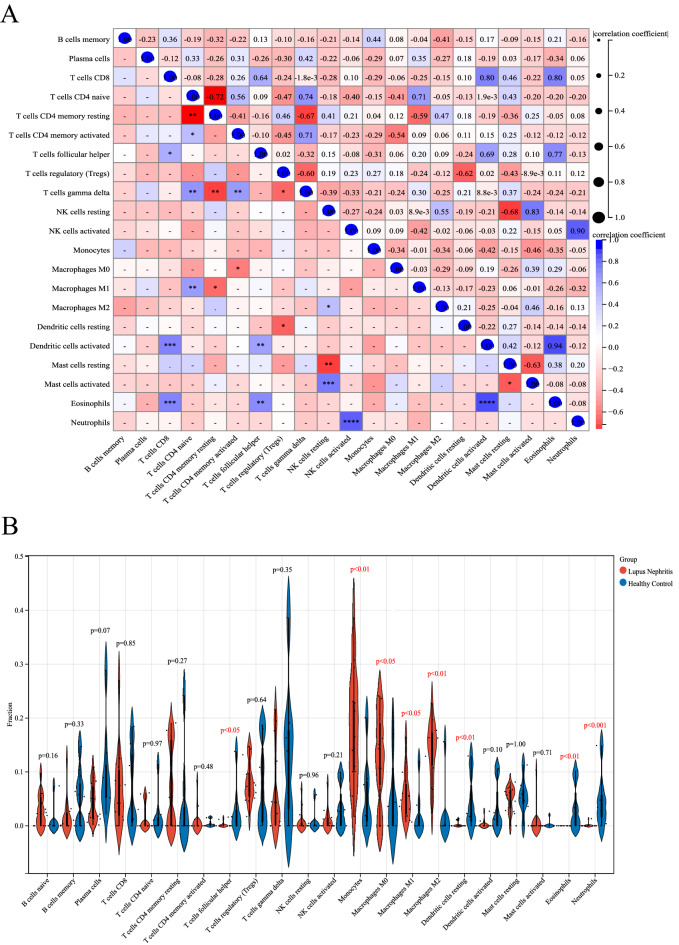
Correlation analysis and bar plot of differences among immune cells in the LN group. (**A**) Correlation analysis. Blue indicates a positive correlation and red indicates a negative correlation; the higher is the absolute value, the stronger is the correlation between immune cells. (**B**) Violin chart showing the proportion of each immune cell type between healthy and LN samples; blue corresponds to healthy samples and red to LN samples, *P* < 0.05.

A positive correlation was detected between CD8 T cells and Dendritic cells activated (r = 0.80), CD8 T cells and Eosinophils (r = 0.80), CD4 naive T cells and γδ T cells (r = 0.74), M1 macrophages and CD4 naive T cells (r = 0.71), as well as Eosinophils and activated dendritic cells (r = 0.94) (Fig. 6A). Instead, a negative correlation was detected between CD4 naive T cells and CD4 memory resting T cells (r = −0.72), CD4 memory resting T cells and γδ T cells (r = −0.67), NK cells resting and Mast cells resting (r = −0.68), T cells regulator (Tregs) and γδ T cells (r = −0.60), as well as Mast cells resting and Mast cells activated (r = −0.63) (Fig. 6A).

Differences in immune infiltrating cells between the kidney tissues of healthy and LN patients were visualized by a violin chart, with statistically significant differences at *P* < 0.05. Monocytes, M1 macrophages, and M2 macrophages were all differentially elevated in the kidney tissues of LN patients, whereas T cells follicular helper, Dendritic cells resting, Eosinophils, and Neutrophils (Fig. 6B).

### Correlation between hub LN-FRGs and immune-infiltrated cells in LN

**Figure 7:**
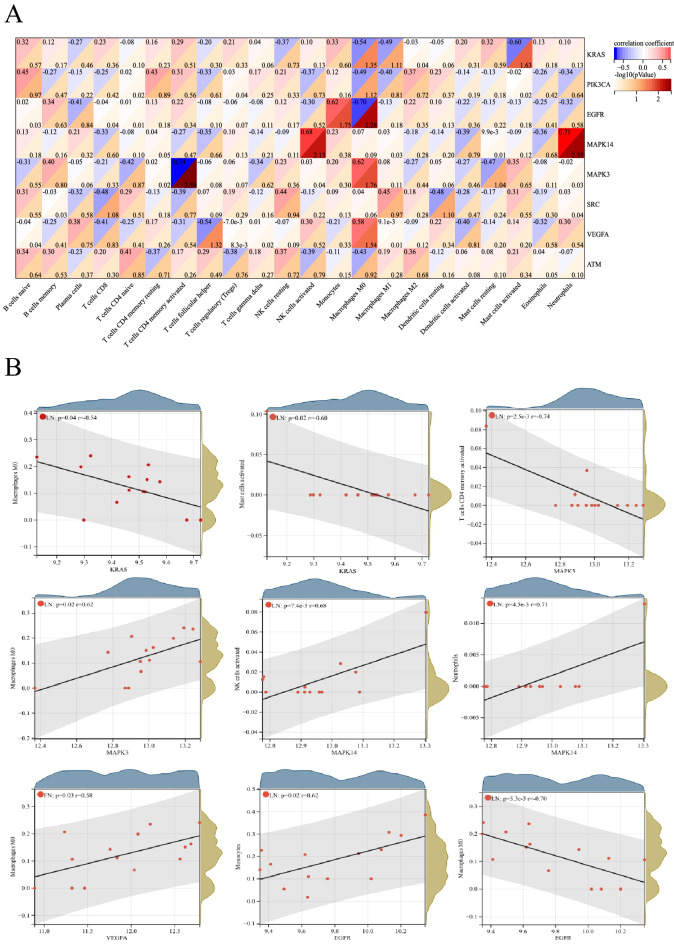
Correlation between hub LN-FRGs and immune-infiltrated cells in LN. (**A**)The darker is the red hue, the smaller is the *P* value. (**B**)The correlation analysis of hub LN-FRGs and immune-infiltrated cells, *P* < 0.05.

The relationship between hub LN-FRGs and immune-infiltrated cells in LN, which differed between LN and control samples, was evaluated by Pearson correlation (Fig. 7). Mast cells activated displayed a negative correlation with *KRAS* (r = −0.60). Monocytes exhibited a positive correlation with *EGFR* (r = 0.62). M0 macrophages displayed a negative correlation with *EGFR* (r = −0.70). NK cells activated showed a positive correlation with *MAPK14* (r = 0.68). Neutrophils exhibited a positive correlation with *MAPK14* (r = 0.71). CD4 memory activated T cells displayed a negative correlation with *MAPK3* (r = −0.74). M0 macrophages exhibited a positive correlation with *MAPK3* (r = 0.62). M0 macrophages displayed a positive correlation with *VEGFA* (r = 0.58). Therefore, these genes were strongly correlated with immune-infiltrated cells in LN.

## Discussion

The pathogenesis of LN is a form of glomerulonephritis that results from the interaction of multiple factors, including environmental and genetic factors^1^. In the present study, a total of 96 DE-FRGs were found to be significantly expressed in LN kidneys, while 8 hub LN-FRGs were also identified, but the extent to which they are involved in the pathogenesis of LN remains to be further investigated. Functional enrichment analysis of GO terms showed that DE-FRGs were mainly enriched in apoptotic process, programmed cell death and ROS. KEGG pathway analysis indicated that the DE-FRGs were mainly enriched in autophagy-animal. Within the PPI network of DE-FRGs, 8 (*KRAS, PIK3CA, EGFR, MAPK14, SRC, MAPK3, VEGFA,* and *ATM*) out of 96 genes had high scores in the 6 algorithms of cytoHubba. GO terms analysis showed that these 8 genes were highly enriched in apoptotic process, cell death and ROS, as well as KEGG that were relaxin signaling pathway and VEGF signaling pathway. Genetic association studies have determined multiple mechanisms regarding the pathogenesis of lupus nephritis, including genetic variants related to alterations in programmed cell death and defects in immune clearance of programmed cell death debris^26^. Although the mechanism of LN has not been fully elucidated, abnormal programmed cell death (e.g. apoptotic process) acts as an important role in its pathogenesis^27^. Since autophagy is essential in maintaining renal cell metabolism and organelle homeostasis, upregulation of autophagic activity may play a role in lupus nephritis to protect and limit kidney injury^28^. Due to the antifibrotic effect of relaxin in an experimental model of chronic kidney disease, it is hypothesized that relaxin may be able to improve the progression of LN^29,30^. VEGF has been shown to be a marker of disease activity in SLE and LN^31^. Both these GO terms and the results of the KEGG pathway suggest that the DE-FRGs or hub LN-FRGs identified in this study may be involved in the progression of LN via the above-mentioned approach.

Overproduction of reactive oxygen species (ROS) in LN has been demonstrated, and inhibition of ROS production reduces the inflammatory response and reduces kidney damage^32,33^. Excessive production of ROS and down-regulation of antioxidant system components also predispose to ferroptosis^34^. *p66shc* belongs to the *SRC* homology and collagen A (*ShcA*) family, a protein associated with ROS generation and capable of induced intracellular ROS production^35^. Thus, *SRC* may influence the process of ferroptosis by causing an imbalance in the antioxidant system or overproduction of ROS, then participating in the pathogenesis of LN. It has been shown that *PIK3CA*, an inhibitor of *PI3Kα*, blocks ferroptosis cell death^36^, but its role in LN has never been reported and further studies are still needed. Somatic mutations in some genes, including *KRAS*, may contribute to the intractability of SLE^37^, and in addition, somatic mutations in *KRAS* cause pediatric Rosai-Dorfman syndrome and SLE^38^. However, as a driver in ferroptosis, the role of *KRAS* in LN is still unknown, and more studies are necessary to confirm whether it is involved in LN through ferroptosis. It has been shown that a gene associated with DNA damage repair, serine/threonine kinase *ATM*, was significantly under-expressed in SLE^39^. *ATM* inhibition rescues ferroptosis by increasing the expression of iron regulatory factors involved in iron storage and export, rather than the typical DNA damage pathway^40^. However, further studies are needed to investigate how *ATM* functions in LN by regulating ferroptosis.

A hallmark of LN is immune cell infiltration. It has been shown that ferroptosis-catalyzed oxide species can also enhance protein- and autoantibody-induced inflammatory transcription factors, leading to the increased matrix, cytokine/chemokine production, and immune cell infiltration, resulting in subsequent increased glomerular permeability and tubulointerstitial inflammation and renal failure interactions^41^. Inefficient clearance of dead cells by dendritic cells and macrophages may lead to disruption of tolerance and result in LN, in part because they provide autoantigens that become components of the immune complexes deposited in the kidney ^42^. Vascular endothelial growth factor A (*VEGFA*), a marker of ferroptosis^43^, has been shown to be highly expressed in the kidney and urine in patients with LN^44^, which suggests that *VEGFA* may be involved in the pathogenesis of LN through ferroptosis. Furthermore, in the present study, *VEGFA* was positively correlated with Macrophage M0, thus it is hypothesized that *VEGFA* decreases the efficiency of Macrophage clearance of ferroptosis cells, which may cause disruption of immune tolerance and lead to LN. In an early study, immunohistochemistry suggested increased *EGFR* expression in about 35% of LN patients^45^. Autoantibodies to the extracellular region of *EGFR* were identified in patients with SLE^46^. The involvement of *EGFR* in the pathogenesis of LN has been demonstrated^47^. In the present study, the level of monocytes infiltration was significantly higher in the kidney tissue of LN patients than in healthy controls. Meanwhile, *EGFR* was positively correlated with monocytes in this study, and it is speculated that *EGFR* may be implicated in the pathogenesis of LN through increased infiltration of monocytes, but whether monocytes are involved in the pathogenesis of LN caused by ferroptosis is unclear. In addition, *EGFR* was negatively correlated with macrophage M0 in the present study, which may be the result of increased *EGFR* leading to decreased macrophages and affecting their ability to clear ferroptosis cells, ultimately exacerbating LN.

Some studies have confirmed the involvement of *MAPK* pathway activation in the pathogenesis of LN^48,49^. Zhang et al. found that myeloid-derived suppressor cells activate *p38*(namely *MAPK14*)*MAPK* signaling by increasing the production of reactive oxygen species in lupus nephritis, which eventually induces podocyte damage^50^. It is therefore possible that *MAPK14* may be implicated in LN pathogenesis by increasing the production of ROS to affect ferroptosis. Zhai et al. found that lipopolysaccharide (LPS) in bacteria could cause significant neutrophils infiltration in the glomeruli of LN, especially around the glomerular membrane zone, while Pyrrolidine dithiocarbamate (PDTC) reduced neutrophils infiltration and the severity of kidney injury by inhibiting NF-κB and p38 MAPK activity^51^. In the present study, *MAPK14* (*p38*) was positively correlated with neutrophils, thus it is hypothesized that *MAPK14* may jointly advance LN occurrence through neutrophils infiltration. It has been found that α2AP induces pro-inflammatory cytokine production in macrophages through the *ERK1*(namely *MAPK3*)/*2* pathway^52^, and combined with the positive correlation between *MAPK3* and Macrophage M0 in the present study, it is therefore hypothesized that *MAPK3* can induce inflammation through macrophage activation thus leading to LN progression. Nevertheless, it remains uncertain how *MAPK3* or *MAPK14* mediates ferroptosis leading to LN through these immune infiltrating cells. Experiments using specifically conditioned immune cell knock-out with these LN-FRGs may help to unravel the potential mechanisms.

The present study presented also some limitations. First, it was based on the GEO database, which is a secondary mining and analysis database of previously published datasets. Hence, the experimental results may differ from the conclusions of previous experiments, most likely due to biased data analysis caused by the small sample size. Second, the CIBERSORT deconvolution algorithm is based on limited genetic data, which may lead to inaccurate results due to different disease predisposing factors and the plasticity of disease phenotypes. Nevertheless, our study may still provide compelling evidence for further research on the potential of the identified immune-infiltrated cells or immune-related genes in ferroptosis for the treatment and diagnosis of LN.

## Conclusion

In summary, overproduction of ROS and abnormal infiltration of immune cells would be implicated in the LN caused by ferroptosis. 8 hub lupus nephritis ferroptosis-related genes (LN-FRGs) which might be good biomarkers of ferroptosis in LN were identified in this study; they include *KRAS, PIK3CA, EGFR, MAPK14, SRC, MAPK3, VEGFA*, and *ATM*. These findings point to the immune response playing an important role in LN caused by ferroptosis via mutual regulation between hub LN-FRGs and immune-infiltrated cells.

## Data Availability

The datasets generated and/or analysed during the current study are available in the [GEO] repository, [https://www.ncbi.nlm.nih.gov/geo/query/acc.cgi?acc=GSE112943].
